# Lateralized Affective Word Priming and Gender Effect

**DOI:** 10.3389/fpsyg.2018.02591

**Published:** 2019-01-11

**Authors:** Ensie Abbassi, Isabelle Blanchette, Bess Sirmon-Taylor, Ana Inès Ansaldo, Bernadette Ska, Yves Joanette

**Affiliations:** ^1^Centre de Recherche, Institut Universitaire de Gériatrie de Montréal & Faculté de Médecine, Université de Montréal, Montréal, QC, Canada; ^2^College of Health Sciences, University of Texas at El Paso, El Paso, TX, United States; ^3^Département de Psychologie, Université du Québec à Trois-Rivières, Trois-Rivières, QC, Canada

**Keywords:** affective priming, hemispheric lateralization, affective (emotional) words, divided visual field, quick (automatic)/slow (controlled) processing, gender effect

## Abstract

Affective priming research suggests that processing of affective words is a quick and short lived process. Using the divided visual field (DVF) paradigm, investigations of the lateralization of affective word processing have yielded inconsistent results. However, research on semantic processing of words generally suggests that the left hemisphere (LH) is the location where rapid processing occurs. We investigated the processing of affective (emotional) words using a combination of the DVF and affective priming paradigms, and four stimulus onset asynchronies (SOAs)—0, 150, 300, and 750 ms. The priming pattern yielded by males (*n* = 32) showed quick priming (at 0-ms SOA) of affective words in the LH; there was slower right hemisphere (RH) priming of affective words (at 750-ms SOA). In females (*n* = 28), both hemispheres were associated with quick priming of affective words (at 300-ms SOA in the LH and at 150-ms SOA in the RH). Results demonstrate the capability of both cerebral hemispheres in the processing of words with affective meaning, along with leading role of the left hemisphere in this process. This is similar to the results of semantic research that suggest access to word meanings occurs in both hemispheres, but different mechanisms might be involved. While the LH seems to prime affective words quickly regardless of gender, gender differences are likely in the RH in that affective word processing probably occurs slowly in males but rapidly in females. This gender difference may result from increased sensitivity to the emotional feature of affective words in females.

## Introduction

There is a large body of research describing an evaluation mechanism in human beings which allows for quick screening of the environment for pleasant and unpleasant stimuli in preparation of appropriate behavioral responses (e.g., Bargh et al., 1992, 1996; Zajonc, 2000; Wentura and Rothermund, 2003). The literature on affective priming provides some of the strongest evidence for this mechanism (for a review, see Fazio, 2001; Klauer and Musch, 2003). This research typically uses the paradigm in which a target word with an affective attribute (e.g., *crime*) is preceded by a prime word with an affective feature (e.g., *gift*); the prime may share the valence (positive or negative) of the target or not. Affective priming is the temporal process by which the evaluation of the target as pleasant or unpleasant is reduced when the emotional valence of the prime and target are congruent rather than incongruent.

The affective priming paradigm was first introduced by Fazio et al. (1986) to show that the affective value of attitude words (positive or negative views of a person, place, thing, or event) is quickly activated in memory upon presentation of a stimulus. In Fazio et al. (1986)’s study, a prime word presented for 200 ms was followed by a target word after a delay of 100 ms. Participants were required to indicate whether the target was *good* or *bad* in meaning. The results showed shorter response times (RTs) to congruent pairs (i.e., negative prime – negative target, positive prime – positive target) than incongruent pairs (i.e., negative prime – positive target, positive prime – negative target). Stimulus Onset Asynchrony (SOA) is the delay elapsed between the onset of the prime and that of the target, and a delay of 300 ms is categorized among short SOA conditions that is not thought to allow participants to develop an explicit expectancy about the target (Neely, 1977, 1991). The priming observed in these conditions was therefore attributed to the quick (or, in one sense, automatic^1^) activation of the affective value of attitude words.

Subsequent studies in the field have not only provided support for the fact that affective priming effect occurs very rapidly (e.g., Hill and Kemp-Wheeler, 1989; Bargh et al., 1992, 1996; Wentura, 1998; Hermans et al., 2001) but have also suggested that the effect is short lived, and diminishes quickly (e.g., Klauer et al., 1997; Hermans et al., 2001). Hermans et al. (2001) examined the time course of affective priming by using the SOAs of 0, 150, 300, and 450 ms. They observed affective priming at the two shorter SOAs (i.e., 0 and 150 ms), but not at the longer two SOAs (i.e., 300 and 450 ms). In other studies, affective priming has been reported at a 300 ms SOA (e.g., Fazio et al., 1986; Bargh et al., 1992, 1996). Hermans et al. (2001) suggested that the activation curve of valence (pleasant/unpleasant) has a very quick onset at the 0-ms SOA but rapidly diminishes such that an SOA of 300 ms probably targets the end of the rapid activation curve.

In most affective priming studies, valenced *words* are used as stimuli. Research examining lateralization of the processing of affective stimuli other than words (i.e., faces, prosody) has emphasized the function of the right hemisphere (RH) in the processing of affective stimuli (e.g., Ley and Bryden, 1979; Borod and Koff, 1989; Adolphs et al., 1996; for review, see Borod, 1992; Demaree et al., 2005). However, research investigating hemispheric contribution to the processing of affective words has yielded more inconsistent results. Some studies provide evidence in favor of the left hemisphere (LH, e.g., Strauss, 1983), others for the RH (e.g., Graves et al., 1981; Collins and Cooke, 2005), and yet others have shown no difference between the contribution of the LH and RH (e.g., Eviatar and Zaidel, 1991; Nagae and Moscovitch, 2002). The paradigm typically used in this research is the divided visual field (DVF) paradigm (Atchley et al., 2003). In the DVF paradigm stimuli are considered to be primarily processed by the LH and RH, based on their presentation to the right visual field (RVF) or the left visual field (LVF), respectively (Chiarello et al., 1990). As affective (valenced) *words* are part of the semantic (language) system for which the role of the LH is unavoidable, the reason for the inconsistent results may be because of the important role that the LH plays in the processing of word stimuli.

Word processing is, however, not restricted to the LH. Studies having examined the respective roles of the two hemispheres for the semantic processing of words suggests that the LH and RH are equivalent information processing systems, each supporting almost all language abilities (for review, see Chiarello, 2003). This is seen most convincingly in the work of Sperry et al. (1969), whose work with patients with split-brains indicated that each hemisphere has the ability to function independently, perform different cognitive processes including memory, perception, and meaning access, and also select appropriate responses to stimuli (Sperry et al., 1969, 1979).

Further, studies looking at the semantic processing of words in normal participants using the DVF priming paradigm, which is a combination of the DVF and priming paradigms, substantiated this hemispheric ability by demonstrating that meanings are processed in both LH and RH, although different mechanisms might be involved (for review, see Chiarello, 1991, 1998). In the DVF priming paradigm, two successive stimuli: the prime and the target (e.g., nurse and doctor), are presented to the LVF or RVF and priming consists in accelerated processing of the target when prime and target are related. Semantic studies using this paradigm and manipulating SOA suggest that quick priming of word stimuli occurs in the LH (Burgess and Simpson, 1988; Abernethy and Coney, 1993, 1996; Koivisto, 1997, 1998). Priming can also occur in the RH, but at longer SOAs, suggesting semantic access re-occurs in the RH, with the involvement of attention (e.g., Brody et al., 1987; Burgess and Simpson, 1988; Koivisto, 1997, 1998; Chan et al., 2006).

Thus, studies looking at the contribution of each hemisphere for the semantic processing of words suggests an important role for the LH in early and quick processing of word stimuli while research on the lateralization of affective word processing intended to show an important role of the RH (e.g., Graves et al., 1981; Strauss, 1983; Eviatar and Zaidel, 1991; Nagae and Moscovitch, 2002; Collins and Cooke, 2005). This RH lateralization is consistent with research on the processing of affective non-word stimuli (e.g., Adolphs et al., 1996; for review, see Borod, 1992; Demaree et al., 2005).

One study has illustrated these contradictory influences using an affective priming paradigm (Brody et al., 1987). Brody et al. (1987) employed the affective priming paradigm with a short SOA of 50 ms and asked their participants to judge whether the target was positive or negative. Instead of reporting priming effects in the LH and RH by comparing RTs to congruent and incongruent pairs, they compared RTs to affective – affective pairs (whether congruent or incongruent) with RTs to neutral – affective pairs^2^. Thus, for example, RTs to positive – positive and negative – positive pairs were treated similarly and compared with RTs to neutral – positive pairs. A detailed inspection of the RT data in Brody et al. (1987)’s study shows affective – affective (including congruent and incongruent) pairs were responded to similarly in the LH and RH; among them, incongruent pairs were responded faster than congruent pairs. This type of priming, which has only been reported in affective priming studies, is called reverse priming (e.g., Glaser and Banaji, 1999; Chan et al., 2006: It is further explained in the discussion). In contrast, neutral – affective pairs were responded to more slowly in the RH (LVF/RH) than in the LH (RVF/LH). Since a comparison between RTs to affective – affective and neutral – affective pairs in the LH (RVF/LH) and RH (LVF/RH) showed faster RTs for the former pairs in the RH than the LH, the authors interpreted the results in favor of the role of the RH in the processing of affective words^3^.

There are two explanations for affective priming effects. Some researchers (e.g., Fazio et al., 1986; Bargh et al., 1992, 1996), drawing on the similarity of the semantic and affective priming paradigms, have proposed a *spread of activation mechanism* (Collins and Loftus, 1975) for affective priming effects whereby the valence of the prime activates a general purpose valence node in semantic memory. The activation of the valence node spreads to words with the same valence and speeds up processing of targets with the same valence. Consequently, targets with negative valence, for instance, become more accessible after presentation of a negative prime than a positive prime^4^.

Since the number of positive or negative concepts in semantic memory is large and the quantity of activation in the network is limited, some researchers (e.g., Wentura, 1999, 2000; Klauer and Musch, 2001) believe that the spread of activation account faces difficulties, at least as an exclusive explanation for affective priming effects. In the so-called *response competition mechanism*, the prime can be evaluated as being the target. Hence, when the prime is congruent to the target, its valence matches the response, but when the prime is incongruent to the target, its valence mismatches the response. Consequently, when prime and target are congruent, response facilitation might occur, whereas when they are incongruent, the resulting interference might slow down responses. Research suggests the involvement of both mechanisms, spread of activation and response competition, in producing affective priming effects (Fazio, 2001; Eder et al., 2012).

In summary, studies having looked at the processing of the emotional valence of words in the cerebral hemispheres have yielded inconsistent results concerning the question of in which hemisphere words with affective or emotional meanings are processed. However, affective priming research has repeatedly indicated that the affective value of words is processed rapidly, suggesting this can be done without attentional resources. Research on semantic processing of words suggests that the LH is the location where rapid processing of word stimuli occurs.

Therefore, the present study was designed to use a combination of the affective priming paradigm and the DVF paradigm, along with different SOAs, to investigate which hemisphere contributes to the processing of affective words. Following Koivisto’s (1997, 1998) and Hermans et al. (2001) studies, four SOAs—0, 150, 300, and 750 ms— were employed. Based on semantic processing research (Burgess and Simpson, 1988; Koivisto, 1997, 1998), we predicted a pattern of quick priming of affective words only in the LH that would diminish quickly. As well, valence has a unique place among word features (e.g., Osgood et al., 1957; Zajonc, 1980; Bargh et al., 1996; Bargh, 1997); research introduces the affective priming as one version of semantic priming (Spruyt et al., 2009). Hence, the leading role of the LH (RVF/LH) in the priming of words with affective (emotional) meaning is expected more than the RH (LVF/RH). We predicted priming at longer SOAs in the RH (LVF/RH).

An additional issue we consider is potential gender differences. Indeed, the emotion literature suggests that, on average, females are more sensitive than males to emotional stimuli such as emotional faces (Thayer and Johnson, 2000; Hall and Matsumoto, 2004; Montagne et al., 2005; Hampson et al., 2006; for a review, see McClure, 2000; Thompson and Voyer, 2014). Importantly for our purpose, there is evidence that this sensitivity occurs automatically (Hatfield et al., 1994; Dimberg, 1997). Based on this, we expected that the pattern of quick affective priming (evidenced by effects prominent at shorter SOAs) to be more evident in female than male participants. This was expected to lead to the contribution of both hemispheres to automatic priming and, therefore, less lateralization in females than males. Because our stimuli are words, this prediction is consistent with the results of a large number of studies that have examined cerebral lateralization for verbal tasks between males and females and have indicated less lateralization in females (for review, see McGlone, 1980; Hellige, 1993; Iaccino, 1993; Hiscock et al., 1994; Voyer, 1996; Voyer, 2011).

## Materials and Methods

### Ethics Statement

This study was approved by the Comité d’éthique de la recherche de l’Institut universitaire de gériatrie de Montréal. All participants provided their written informed consent to participate in this study.

### Design

A 2 (congruency: congruent vs. incongruent) × 2 (visual field: RVF vs. LVF) × 4 (SOA: 0 vs. 150 vs. 300 vs. 750 ms) × 2 (gender: males vs. females) mixed factorial design was used. The only between-participant factor was gender. The primary dependent variable was reaction time; percent correct scores were also measured. It needs to be mentioned here that valence is not a factor examined in affective priming studies (the reader is referred to publications in affective priming literature); priming effects cannot be decomposed into positive and negative parts: priming effects for positive targets and negative targets are confounded by main effects^5^.

### Participants

A total of 62 students from English-language universities in Montreal between the ages of 20 and 35 participated on a voluntary basis (with compensation). Data from 2 participants were randomly discarded because a balanced number of participants in the 4 SOA conditions was needed. Although PANAS (i.e., Positive and Negative Affect Scale: Watson et al., 1988) scores of three participants (two females and one male) implied they suffered from depressed mood at the time of the experiment, their data were not removed from analysis in order to maintain a balanced number of participants in the 4 SOA conditions. That is because the data of five more participants (i.e., 2 females and 3 males) would have had to be removed if we had excluded these three participants. Thus, we decided to maintain these participants in the analysis. (It is worth mentioning that excluding these three participants from the analysis did not change the results of the study. Stronger results were actually observed; participants with depressed mood responded more slowly to stimuli).

Thus, analyses were run with the data of 32 male and 28 female participants; their mean age was 25.4 years (*SD* = 4.1) for males and 24.5 years (*SD* = 3.9) for females. All were right-handed native English speakers, without any past history of neurological or psychiatric disease, and with normal or corrected to normal vision. Their handedness scores, as assessed by the Edinburgh Handedness Inventory (Oldfield, 1971), were 91 (*SD* = 14) for males and 97 (*SD* = 6) for females. With the aim of controlling for the possible impact of hormonal fluctuations (i.e., the possible effect of a decrease in LH activation in the premenstrual phase: Altemus et al., 1989; Alexander et al., 2002), female participants were tested in the postmenstrual phase of their menstrual cycles (days 5–17, with a mean of 11). In addition, females who were taking estrogen for birth control were excluded from the study.

### Stimuli

The stimuli (see Appendix) were selected from the Affective Norms for English Words (ANEW) list (Bradley and Lang, 1999), which is a standard set of 1,034 English words that have been characterized along the dimensions of valence (from negative to positive, with a range of from 1 to 9) and arousal (from low to high, with a range of from 1 to 9). In the present study, 48 positive and 48 negative words with high arousal ratings (5–9) were selected from the words with valence ratings of 1 to 3 (negative words) and 7 to 9 (positive words). Then 48 congruent prime-target pairs [i.e., positive–positive (P–P) and negative–negative (N–N)] were constructed. The prime and target of each congruent pair had either negative or positive valence but were not semantically related. The lack of semantic relatedness, which implies that the prime and target in each pair were associated only by their emotional feature, was confirmed by performing a *word association task* (Rossell and Nobre, 2004) in which we asked 21 of the participants at the end of their experimental session to list three words that they immediately associated with each prime word. Therefore, each prime was paired with about 63 associations, and the target was never among those associations.

The positive and negative prime sets and target sets were carefully matched according to grammatical category, imageability, concreteness, and word length (Medical Research Council psycholinguistic database: Coltheart, 1981). Stimuli were used in English either as nouns (e.g., *song, crime*) or as both nouns and verbs (e.g., *rescue, assault*). All words were 3 to 7 letters long, with the mean length of prime and target sets being 5.42 and 5.29, respectively. Frequency ratings were slightly different, with mean ratings for P–P pairs and N–N pairs being 72.67 and 40.25, respectively. This could not be avoided since we did not have a large enough pool of emotional words from which to choose a frequency-balanced subset; in fact, we had to match the stimuli in terms of seven different features (i.e., valence, arousal, concreteness, grammatical category, imageability, word length, and frequency). Nevertheless, we strove to have the closest frequency rating possible for the positive and negative sets. It should be mentioned that this difference is a normal attribute of language: positive words are more frequent than negative words (Osgood et al., 1957). Hence, an imbalance is unavoidable in studies that use a larger stimulus set (e.g., Wurm and Vakoch, 1996; Scott et al., 2009).

The congruent prime-target pairs were re-paired to form 48 incongruent positive–negative (P–N) and negative–positive (N–P) pairs. To counterbalance for congruency and visual field of presentation, the congruent and incongruent pairs were parsed into two sub-lists, each with 24 congruent pairs (12 in each visual field) and 24 incongruent pairs (12 in each visual field). Mirror-image sub-lists were prepared by reversing the visual field of presentation, resulting in four different test lists. Within each list, every prime-target pair was unique. Assignment of SOAs to the test lists was counterbalanced. Therefore, there was a total of 48 prime-target pairs in each test list and each participant received all four test lists, one for each SOA condition (i.e., 0, 150, 300, and 750 ms), for a total of 192 pairs for the four SOA conditions. The trials in each block were preceded by three buffer trials. In each block, half of the pairs were presented in the RVF and half in the LVF (i.e., RVF prime – RVF target, LVF prime – LVF target). A practice set, which included none of the stimuli from the experimental lists, consisted of four blocks of 16 trials, one for each SOA, which introduced the SOAs that would be presented in the following blocks. Stimuli were presented in white uppercase against a black background, subtending 1.6–4° and 0.9° of horizontal and vertical visual angles, respectively (Koivisto, 1997).

### Procedure

Before running the practice trials, participants completed three paper-and-pencil questionnaires: a participant information form, the PANAS (Watson et al., 1988), and the Edinburgh Handedness Questionnaire (Oldfield, 1971). When this was done, the participant sat 60 cm away from the computer screen; to maintain a constant viewing distance, a chin rest was used. All instructions were explained to the participant by the experimenter, who also monitored eye movements using a video camera attached to a television (McKeever, 1986) during practice trials. Participants were reminded if any deviation from the fixation point was noted. Participants were instructed to always focus on the central fixation point and to make their judgments based on their peripheral vision. They were told that this would yield optimal performance since the location of both prime and target was determined randomly. The training lasted for 40–45 min.

The main experiment started when the participant was capable of responding parafoveally (the procedure described above) and lasted for 20–25 min. The experimenter continued to monitor participants’ eye movements throughout the experiment, though; eye movement did not occur during the main experiment. Stimulus presentation, data collection, and timing were controlled by E-Prime 1.0 software. The session began with trials that provided opportunity for practice, followed by one of the experimental blocks. The presentation order of the four blocks was counterbalanced across participants. Participants were allowed to rest briefly between blocks.

**FIGURE 1:**
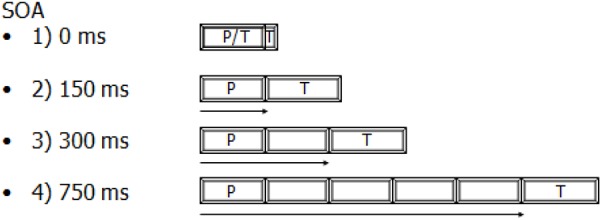
A comparison between the duration of the presentation of the prime (P: 150 ms) and the target (T: 180 ms) and also the delay elapsed between their presentations in the four SOA conditions.

In each trial, a fixation point was presented at the center of the screen. Each trial began with an alerting tone; after a 700-ms period, the prime was displayed in the RVF or LVF for 150 ms. A block’s SOA determined whether 0, 150, 300, or 750 ms elapsed between the onset of the prime and the onset of the target. Targets were consistently presented for 180 ms (Figure 1 compares the duration of the presentation of the prime and the target and also the delay between their presentations in the four SOA conditions). The next trial was initiated 1 s after the participant’s response. Alternatively, if the participant did not respond within 1600 ms, an error was documented and the next trial was initiated after 1 s. The target was always underlined (Hermans et al., 2001). This was important, especially at the 0-ms SOA, for which the presentation of the two stimuli overlapped. Trials were presented in pseudo-randomized order with no more than three successive trials in the same visual field. The prime and target were presented to the same visual field. However, the location of the presentation of the target was also unpredictable. Indeed, the target was randomly displayed above or below on the right or left side of the prime (i.e., four possible locations). So, re-fixating on the prime location was impossible. Participants were informed that the underlined word would either be presented simultaneously with another word or follow it.

Participants were directed to focus on the central fixation point. They were instructed to read both words but to respond only to the underlined word. Participants were required to decide as quickly and accurately as possible if the target word was *pleasant* or *unpleasant*. Responses were registered by simultaneously pressing the “c” and “m” keys of a keyboard placed symmetrically at the midline for pleasant words and the “d” and “k” keys for unpleasant words. The faster of the two responses was taken as the RT for that trial^6^ (Collins and Cooke, 2005; Weems and Zaidel, 2005). In addition to RT, percent accuracy scores were measured.

## Results

No transformation for RT data was performed; the data were inspected and found to be distributed normally, as confirmed by *Z*-values statistical test. We decided not to use data trimming either, in order not to remove a significant portion of the data. Therefore, RT data analysis performed using participants’ average RTs for each condition involved in the experiment. We only included trials when participants provided correct responses.

The RTs were compared using a 2 × 2 × 4 × 2 mixed ANOVA with congruency (congruent vs. incongruent), visual field (RVF vs. LVF), and SOA (0 vs. 150 vs. 300 vs. 750 ms) as within-participant factors and gender (males vs. females) as between-participant factor. This showed a significant main effect of gender, *F*(1,58) = 4.68, *p* = 0.035, $\eta_{p}^{2}$ = 0.08. Overall, females’ RTs were faster than males’ RTs (574 ms vs. 620 ms). There was a main effect of visual field, *F*(1,58) = 15.27, *p* < 0.01, $\eta_{p}^{2}$ = 0.21, showing that responses to target words in the RVF (590 ms) were faster than responses to target words in the LVF (604 ms). Main effect of SOA was also significant, *F*(3,174) = 22.29, *p* < 0.01, $\eta_{p}^{2}$ = 0.28. *Post hoc* comparisons showed significant differences at an alpha level of 5% between RTs at the SOA of 0 ms (642) and each of the 150-ms (585), 300-ms (570), and 750-ms (592) SOAs.

**FIGURE 2:**
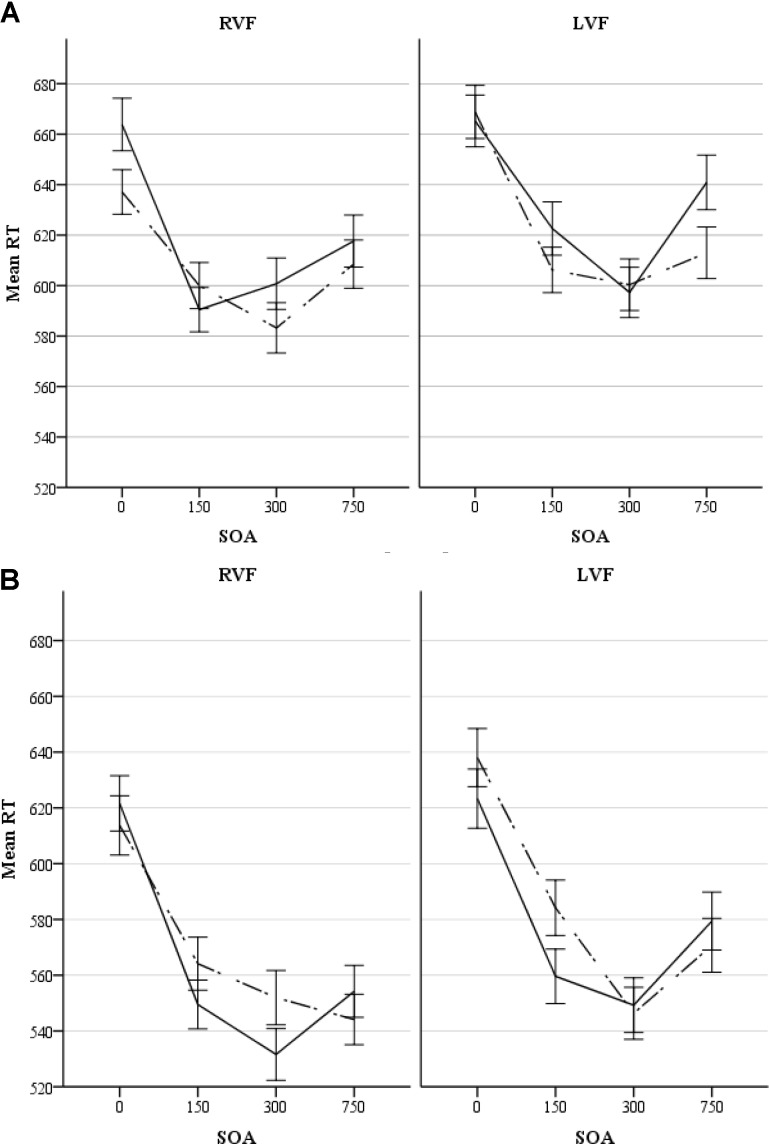
**(A)** Response times (ms) to congruent and incongruent pairs in each visual field as a function of SOA in male participants. The solid line and the dotted line represent congruent and incongruent pairs, respectively. Errors bars represent standard errors. **(B)** Response times (ms) to congruent and incongruent pairs in each visual field as a function of SOA in female participants. The solid line and the dotted line represent congruent and incongruent pairs, respectively. Errors bars represent standard errors.

There was an almost significant^7^ four-way interaction of all the factors involved in the experiment (i.e., congruency × visual field × SOA × gender), *F*(3,174) = 2.36, *p* = 0.073, $\eta_{p}^{2}$ = 0.04. This interaction suggests that the pattern of priming in the two visual fields was modulated by SOA in one of the genders. Tables 1A,B show average RTs and percent accuracy scores for congruent and incongruent pairs, along with priming effects at each SOA in the RVF and LVF in males and females, respectively. Figures 2A,B display priming effects in the visual fields as a function of SOA in males and females, respectively. Table 2 compares RTs to congruent pairs and also incongruent pairs in each visual field as a function of SOA between male and female participants.

**Table 1A:** Average response times (ms) and accuracy (percent correct) for congruent and incongruent pairs in each visual field as a function of SOA (SD) in male participants.

	SOA							
	
	0 ms		150 ms		300 ms		750 ms	
				
	RT	Accuracy	RT	Accuracy	RT	Accuracy	RT	Accuracy
RVF								
Congruent	663 (104)	85 (11)	593 (82)	88 (11)	601 (100)	88 (10)	619 (109)	90 (9)
Incongruent	637 (92)	87 (12)	604 (98)	86 (11)	583 (102)	87 (14)	611 (103)	90 (9)
Priming	-26^∗^	2	12	-2	-18	-1	-8	0
LVF								
Congruent	666 (105)	81 (11)	623 (104)	84 (13)	598 (107)	85 (13)	640 (115)	91 (11)
Incongruent	668 (111)	81 (15)	607 (87)	83 (13)	597 (95)	87 (11)	612 (102)	87 (10)
Priming	2	0	-15	-1	-1	2	-28^∗^	-4


*^∗^significant priming effects (p < 0.05).*

**Table 1B:** Average response times (ms) and accuracy (percent correct) for congruent and incongruent pairs in each visual field as a function of SOA (SD) in female participants.

	SOA							
	
	0 ms		150 ms		300 ms		750 ms	
				
	RT	Accuracy	RT	Accuracy	RT	Accuracy	RT	Accuracy
RVF								
Congruent	620 (100)	86 (11)	550 (99)	89 (11)	531 (95)	91 (9)	555 (96)	91 (9)
Incongruent	613 (110)	85 (16)	561 (102)	86 (15)	557 (109)	90 (10)	546 (97)	91 (10)
Priming	-7	-1	11	-3	26^∗^	-1	-9	0
LVF								
Congruent	628 (122)	82 (17)	558 (100)	89 (9)	548 (96)	92 (8)	579 (90)	88 (10)
Incongruent	639 (121)	83 (15)	584 (100)	87 (10)	549 (81)	87 (14)	573 (90)	87 (15)
Priming	11	1	25^∗^	-2	1	-5	-6	-1


*^∗^significant priming effects (p < 0.05).*

**Table 2:** Results of *post hoc* comparison of RTs of male and female participants to congruent and incongruent pairs in each visual field as a function of SOA.

Conditions	Gender	Mean	*SD*	df	*F*	Sig.	$\eta_{p}^{2}$
Congruent pairs in RVF at SOA of 0	M	663	104	(1, 58)	2.612	0.112	0.04
	F	620	100				
Incongruent pairs in RVF at SOA of 0	M	637	92	(1, 58)	0.829	0.366	0.01
	F	613	110				
Congruent pairs in RVF at SOA of 150	M	593	82	(1, 58)	3.375	0.071	0.06
	F	550	99				
Incongruent pairs in RVF at SOA of 150	M	604	98	(1, 58)	2.764	0.102	0.05
	F	561	102				
Congruent pairs in RVF at SOA of 300	M	601	100	(1, 58)	7.771	0.007	0.12
	F	531	95				
Incongruent pairs in RVF at SOA of 300	M	583	102	(1, 58)	0.913	0.343	0.02
	F	557	109				
Congruent pairs in RVF at SOA of 750	M	619	109	(1, 58)	5.694	0.02	0.09
	F	555	96				
Incongruent pairs in RVF at SOA of 750	M	611	103	(1, 58)	6.28	0.015	0.10
	F	546	97				
Congruent pairs in LVF at SOA of 0	M	666	105	(1, 58)	1.663	0.202	0.03
	F	628	122				
Incongruent pairs in LVF at SOA of 0	M	668	111	(1, 58)	0.909	0.344	0.02
	F	639	121				
Congruent pairs in LVF at SOA of 150	M	623	104	(1, 58)	5.911	0.018	0.09
	F	558	100				
Incongruent pairs in LVF at SOA of 150	M	607	87	(1, 58)	0.959	0.332	0.02
	F	584	100				
Congruent pairs in LVF at SOA of 300	M	598	107	(1, 58)	3.548	0.065	0.06
	F	548	96				
Incongruent pairs in LVF at SOA of 300	M	597	95	(1, 58)	4.222	0.044	0.07
	F	549	81				
Congruent pairs in LVF at SOA of 750	M	641	115	(1, 58)	5.149	0.027	0.08
	F	579	90				
incongruent pairs in LVF at SOA of 750	M	612	102	(1, 58)	2.485	0.12	0.04
	F	573	90				
$\eta_{p}^{2}$: Partial Eta Squared							


Performing two 2 × 2 × 4 repeated-measures ANOVAs with congruency (congruent vs. incongruent), visual field (RVF vs. LVF), and SOA (0 vs. 150 vs. 300 vs. 750 ms) for each gender showed an almost significant three-way interaction involving congruency, visual field, and SOA in males, *F*(3,93) = 2.37, *p* = 0.076, $\eta_{p}^{2}$ = 0.07, which indicates that the pattern of priming in the two visual fields was modulated by SOA in males (the results of the decomposition of this interaction is provided in the next paragraph). In females this interaction which involves hemisphere (congruency × visual field × SOA) was not significant, *F*(3,81) = 1.59, *p* = 0.197, $\eta_{p}^{2}$ = 0.06, but two-way congruency × SOA interaction was almost significant, *F*(3,81) = 2.36, *p* = 0.077, $\eta_{p}^{2}$ = 0.08. Examining the simple effects of congruency within each SOA condition showed 18 ms of priming at the 150-ms SOA, *F*(1,27) = 5.68, *p* = 0.024, $\eta_{p}^{2}$ = 0.17, and 14 ms of priming at the 300-ms SOA, *F*(1,27) = 2.03, *p* = 0.17, $\eta_{p}^{2}$ = 0.07. Priming that emerged at the SOAs of 0-ms and 750-ms was negligible (*F*s < 1).

Because this was directly related to our hypotheses, we examined the simple effect of congruency at each SOA condition within the RVF and LVF in males and females. In males, comparison between RTs to congruent pairs and RTs to incongruent pairs demonstrated that in the RVF (LH), there was a 26-ms significant reverse priming effects at the 0-ms SOA, *F*(1,58) = 5.99, *p* = 0.017, $\eta_{p}^{2}$ = 0.09. Priming was not significant at 150-ms (*F* < 1.2), 300-ms, *F*(1,58) = 2.2, *p* = 0.147, $\eta_{p}^{2}$ = 0.036, and 750-ms SOAs (*F*s < 1). In the LVF (RH), there were not significant priming effects at the 0-ms SOA (*F* < 1), 150-ms SOA, *F*(1,58) = 1.77, *p* = 0.19, $\eta_{p}^{2}$ = 0.03, and 300-ms SOA (*F* < 1). However, A 28-ms significant reverse priming effects occurred at the SOA of 750 ms, *F*(1,58) = 6.88, *p* = 0.011, $\eta_{p}^{2}$ = 0.11. Altogether the pattern of priming in males suggests an earlier priming effect in the LH and a later priming effect in the RH.

In females, comparison between RTs to congruent pairs and RTs to incongruent pairs indicated that in the RVF (LH), the only effect was a 26-ms significant priming effects at the 300-ms SOA, *F*(1,58) = 4.073, *p* = 0.048, $\eta_{p}^{2}$ = 0.07. There was no priming at the 0-, 150-, and 750-ms SOAs (*F*s < 1.1). In the LVF (RH), there was only a 25-ms significant priming effects at 150-ms SOA, *F*(1,58) = 4.148, *p* = 0.046, $\eta_{p}^{2}$ = 0.07, whereas priming effects at 0-ms SOA, 300-ms SOA, and 750-ms SOA were negligible (*F*s < 1.1). Thus, altogether the data from female participants did not show priming at the long SOA (750-ms SOA) in any hemisphere: the involvement of both the LH and RH in rapid priming effects occurred.

Accuracy was not the main variable in our study. However, we examined accuracy scores in order to rule out speed-accuracy trade-off. To do this, we ran a Pearson correlation test with the RT and accuracy scores of each experimental condition (16 tests, in overall). These tests showed no correlation between the RT and accuracy scores at any of the experimental conditions (all *p*s > 0.20).

Moreover, running the same ANOVA as the one used for RT data (2 × 2 × 4 × 2 mixed ANOVA) with accuracy as a dependent variable did not show a gender effect, *F*(1,58) = 0.472, *p* > 0.05, $\eta_{p}^{2}$ = 0.00. Namely, there was no difference between males’ accuracy scores (86.3%) and females’ accuracy scores (87.6%). Main effect of visual field was significant, *F*(1,58) = 10.78, *p* < 0.01, $\eta_{p}^{2}$ = 0.16, showing that responses were more accurate to the RVF stimuli (88%) than to the LVF stimuli (86%). Congruency did not have a statistically significant effect (*F* < 2.1), but the main effect of SOA was significant, *F*(3,174) = 15.20, *p* < 0.01, $\eta_{p}^{2}$ = 0.21. *Post hoc* comparisons showed significant differences at an alpha level of 0.05 between percent accuracy scores at the SOA of 0 ms (83.5%) and each of the 150-ms SOA (86.5%), the 300-ms SOA (88.3%) and 750-ms SOA (89.4%). None of the two-way, three-way, and four-way interactions did show reliable effects. Hence, the similarity between RT and accuracy results, particularly with regard to SOA (e.g., slower RTs and decreased accuracy at 0 ms) and visual field (e.g., faster RTs and higher accuracy for RVF stimuli than for LVF stimuli) makes the possibility of a speed-accuracy trade-off in the data of the study unlikely.

## Discussion

The present study was designed to investigate the lateralization of affective word priming, which is considered a rapid and short lived process. A DVF affective priming paradigm was used examining four different SOAs: 0, 150, 300, and 750 ms. Important effects of gender emerged. Consistent with results of research on the processing of emotional facial expressions, our results suggested that females were faster than males in responding to affective words. There were also gender differences in terms of priming. For males, priming of affective words occurred rapidly and it was lateralized to the LH (RVF/LH). There was some evidence of affective priming in the RH (LVF/RH) but this occurred later. In females, there was less lateralization. Both hemispheres showed evidence of rapid affective priming: at the SOA of 300 ms in the LH (RVF/LH) and at the SOA of 150 ms in the RH (LVF/RH). In comparison between the priming effects of the LH and RH, priming did not occur at the longer SOA in the LH (in both genders). Thus, the LH seems to prime emotional words quickly regardless of gender. In the RH, on the other hand, priming occurred slowly in males but rapidly in females.

We first discuss the results observed in males, followed by those of females including likely reasons for the gender effect. In light of our results, we also discuss mechanisms involved in producing affective priming effects.

### Pattern of Priming in Males

In males, a pattern of quick priming in the LH (RVF/LH) vs. slow priming in the RH (LVF/RH) was found. The absence of priming in the RH at the short SOA condition likely suggests that the RH is not sensitive to the affective relationships between words early in processing. The same way that priming appears quickly in the LH, it also diminishes quickly: at the 150-ms and 300-ms SOAs, reliable priming was not detected in this hemisphere. The observed pattern of priming seems compatible with the pattern suggested by semantic research (Koivisto, 1997, 1998; for a review, see Abbassi et al., 2011). This study suggests that early and quick semantic priming results from the processing sustained by the LH and that processing is then shifted to the RH, which leads to observing priming at a longer SOA^8^. This latter form is often termed controlled or attentional priming (Neely, 1977, 1991). The results observed in males in our study is largely consistent with this pattern and suggests that affective value of words is accessed within each hemisphere in a way consistent with more general semantic processing: first in the left, and then in the right hemisphere.

The results are consistent with the more general idea that emotional stimuli are processed quickly and without intent (Fazio et al., 1986). The time course of affective priming starts earlier than semantic priming (compare with: Burgess and Simpson, 1988; Abernethy and Coney, 1993, 1996; Koivisto, 1997, 1998). However, the delayed activation of emotional words in the RH at the 750-ms SOA, in conjunction with the lack of priming in the LH at the same SOA, seems to suggest that affective word activation can re-occur in a controlled mode by directing attention to the content of these words and that this process is driven by the RH. So, when sufficient time has elapsed and the meaning is suppressed in the LH, access is possible through the RH. This may imply that the hemisphere that is dominant over early aspects of emotional word processing is not dominant over later and controlled aspects. One possible way of interpreting these data is because the level of emotional word processing is different in the two hemispheres. That is, the LH has a quick but superficial access to the meaning of emotional words whereas access in the RH is slow but deep in that emotional properties have a determining role (for a review, see Abbassi et al., 2015).

Altogether the pattern of results in males suggests rapid processing of affective words in the LH with evidence of later further processing of affective words in the RH.

### Pattern of Priming in Females and Possible Reasons for the Gender Effect

Our study demonstrated that females respond to affective words faster than males. This result is consistent with results of emotion studies examining the processing of emotional faces in males and females (Hall and Matsumoto, 2004; Montagne et al., 2005; Hampson et al., 2006; Thayer and Johnson). In females, priming effects were observed at 150- and 300-ms SOAs, both within the range indicating rapid priming (Neely, 1977, 1991). Contrary to males for whom rapid affective priming was lateralized to the LH, there was evidence of rapid priming in both hemispheres which seems to support the quick activation of emotional words in females.

To explain gender differences in emotion processing, some studies suggest that females are more likely to feel the valence (i.e., pleasantness and unpleasantness) indicated by emotional stimuli when they respond to these stimuli (e.g., Burton and Levy, 1989; Van Strien and Van Beek, 2000; Rodway et al., 2003). There is also evidence that this increased sensitivity results from quick and automatic processes (Hatfield et al., 1994; Dimberg, 1997). Accordingly, it is possible that with the high arousing words that we used in our study, the feeling of the related valence instigated by an emotional stimulus was the mechanism that caused females to reach a decision regarding the target (for a review, see Ley and Strauss, 1986).

In a meta-analytic review of the studies that compared the processing of emotional faces in males and females from infancy through adolescence, McClure (2000) argues that the development of emotional facial perception goes hand in hand with the development of language. McClure believes that mothers are more emotionally expressive toward their female than their male children. By using more varied emotional language in conversations with their daughters, mothers teach them to be more sensitive to the emotions of people around them.

Therefore, the results show less lateralization in females than males in the processing of words with emotional meaning.

### Mechanisms Involved in Affective Priming

Though we discuss the findings overall in terms of priming, it must be noted that our results suggest both spread of activation and response competition mechanisms are involved in producing affective priming effects (Fazio, 2001; Eder et al., 2012). We observed evidence of reverse priming effects at 0- and 750-ms SOAs (for reverse priming at a longer SOA: see Klauer et al., 1997, 2009, experiment 3). In reverse priming faster RTs occur to incongruent than congruent pairs (e.g., Glaser and Banaji, 1999; Chan et al., 2006); this reverse effect is evidence that typically supports the response competition account, i.e., a competition between the valence of the prime and that of the target. Although Wentura and Rothermund (2003) believe that reverse priming occurs when accuracy is emphasized, accuracy was not in the center of our instruction. Indeed, we asked participants to be both accurate and quick, and not to sacrifice one for the other.

On the other hand, some researchers argue that reverse priming occurs when the prime becomes salient, such as when it is highly arousing (Glaser and Banaji, 1999) or frequent (e.g., Chan et al., 2006). In this condition, participants probably recognize the potential effect of the prime to bias their judgment regarding the target and, consequently, attempt to ensure that the response is provided to the right item: the target. As it is easier to distinguish the target from the prime in the case of incongruent pairs, participants respond faster to incongruent pairs. In contrast, a double check will result in extended RTs for congruent pairs (Wentura, 2000).

While it seems that available accounts of affective priming effects have difficulties explaining the occurrence of reverse priming at short SOAs (Klauer et al., 2009), we provide a tentative explanation for our finding at 0-SOA condition. Presumably, at an SOA of 0 ms the valence of the target and the prime are first activated simultaneously and then compared with regard to their being pleasant or unpleasant (see Wentura and Rothermund, 2003). This serial process (parallel coactivation of the prime and the target and then their comparison) would result in longer RTs than when there is a delay between the presentation of the prime and the target (Hermans et al., 2001). And in our study, we obtained the slowest RTs at the 0-ms SOA. These explanations should wait for further confirmation, though.

Most likely, at 750-ms SOA, two successive decisions are made by the participant, the first concerning the prime and the second the target. The participant has sufficient time to identify the valence of the prime before the target appears. Accordingly, reverse priming occurs because, when the prime is encoded with different information, distinguishing it from the target becomes easier. Thus, discrepancy speeds up the decision, whereas in the case of identical valences for prime and target, the need for a double check results in longer RTs (Klauer et al., 1997; Wentura and Rothermund, 2003).

At 150- and 300-ms SOAs (e.g., Fazio et al., 1986; Bargh et al., 1992, 1996), a pattern of typical priming, with shorter RTs for congruent than incongruent pairs, is more likely. At these SOAs, spreading of activation is probably the mechanism underlying the priming effects. Presumably, the delay between presentation of the prime and the target causes the participant to get ahead in accessing the representation of the prime; consequently, spreading of activation to words with a congruent valence facilitates responses to these pairs.

## Conclusion

The present study suggests that the time course of processing of affective words is different in the cerebral hemispheres, and that this differs importantly based on gender. There is important lateralization in males, with rapid affective processing based on the LH (RVF/LH) vs. more controlled processing based on the RH (LVF/RH). In females, a pattern of activation in which both hemispheres share the quick activation of affective words is observed. This may result from increased sensitivity to the emotional feature of stimuli.

## Author Contributions

EA conception and development of the idea and design, performed the experiments, analyzed the data, wrote the manuscript, and discussed the results and implications with co-authors. IB development of the idea, substantial feedback on data analysis and content, discussion of the results and implications, comments on the manuscript. BS-T, AA, and BS discussion of the results and implications and comments on the manuscript. YJ substantial contribution to the development of the idea, design, and data analysis, feedback on content and overall paper for submission.

## Conflict of Interest Statement

The authors declare that the research was conducted in the absence of any commercial or financial relationships that could be construed as a potential conflict of interest.

## Footnotes

1 In social psychology in which affective priming paradigm is used to measure attitudes and prejudices and to predict behavior, automaticity is suggested to be diagnosed by the presence of features such as unintentional, uncontrollable, unconscious, efficient, and rapid processing (Moors and De Houwer, 2006). At present, however, there is little agreement about which features are central for automaticity (for review, see De Houwer et al., 2009). Klauer et al. (2009), for example, uses the term *spontaneous* for this type of processing. In this paper, we decided to use terms like rapid, quick, or fast, instead of automatic, because we can safely make conclusions about time, but not more generally about automaticity. Nevertheless, we should consider one other important fact related to *automaticity*. Recent emotion research has differentiated between two notions related to automatic processing: *strongly automatic* and *partially automatic*. Strongly automatic implies independence from top-down factors, for example, as is assumed to occur during subconscious processing; this type of automatic processing seems to be supported by subcortical pathways. By contrast, partially automatic refers to that type of involuntary processing in which some amount of processing is obligatory, but still modifiable by attention (e.g., for review, see Pessoa, 2005). The type of automatic (rapid) processing applicable to the subject of this study is mostly equivalent to the latter definition of automaticity.

2 The reason neutral – affective pairs are used is for measuring the amount of facilitation vs. inhibition in producing priming effects (e.g., Hermans et al., 2001). In later and recent priming research, however, this comparison is eliminated (e.g., Koivisto, 1997, 1998; Grose-Fifer and Deacon, 2004; Kandhadai and Federmeier, 2010), perhaps because it is not very informative for the purpose of this research.

3 The RH hypothesis holds that the RH is dominant in the processing of *emotional* stimuli. This implies that the LH is dominant in the processing of *neutral* stimuli. This hypothesis has mainly received support from research investigating the processing of *non-verbal* emotional stimuli (e.g., emotional facial expression, prosody). Brody et al.’s study as one of the first studies conducted in the field of emotional *word* processing intended to provide evidence for the dominant role of the *RH* in *emotional word* processing. Our hypothesis does not deny that the RH is capable of emotional word processing but it hold that the LH is also involved: their difference is due to their time course of activation. In Brody et al.’s results, in the direction of the RH hypothesis, neutral pairs were responded to more slowly in the RH than the LH. Considering that RTs to emotional pairs were similar in the LH and RH, the pattern of responding to neutral words caused the difference between RTs to neutral pairs and RTs to emotional pairs to be larger and significant in the RH. This was considered by Brody and colleagues as evidence supporting the role of the RH in emotional word processing. Our point here is that in the affective priming paradigm a comparison must be made between RTs to congruent pairs and RTs to incongruent pairs in order to see whether activation (i.e., significant priming) occurs within a given hemisphere. Neutral pairs are not the main point of reference in the priming paradigm.

4 In affective priming literature, pronunciation task is typically used for the discussion concerning the mechanism that underlies the affective priming effects. Indeed, affective priming of pronunciation task is considered as evidence for the spread of activation mechanism being the underlying mechanism of affective priming effects. While some studies have shown reliable priming with this task (Bargh et al., 1996; Bargh, 1997), others have failed to show this effect (De Houwer et al., 1998; Klauer and Musch, 2001; Spruyt et al., 2004).

5 “It is important to realize that one cannot interpret separate priming effects for positive and negative stimuli because those separate effects are confounded with a main effect. For instance, the difference between P–P and N–P could be due to priming (impact of prime on target responding) but also to a main effect of prime (e.g., always slower after negative primes). Likewise, the difference between P–P and P–N could be due to priming or to a main effect of target (e.g., always faster responding to positive targets). Only the interaction can be interpreted as a priming effect. This priming effect can be recoded as a main effect of congruence. Analyses with congruence as a factor are also more simple. For those two reasons, analyses with congruence became the norm” (Professor Jan De Houwer, personal communication, December 11, 2017).

6 The rationale for two-finger responses is: “each index finger is predominantly connected to the contralateral hemisphere. If only right index finger responses are used, this will advantage only the LH whereas the two-index-finger responses mean that each hemisphere has an equal chance of responding first. Therefore, we are more likely to be able to identify the RH’s contributions” (M. A. Collins, personal communication, January 3, 2011).

7 It is to be noted that in the present study all recorded RTs were included in the analysis, unlike other studies in the field in which some extreme RTs were excluded from the analysis, thus, this probably contributing to the marginally significant results in the present study.

8 In lateralization studies, slow mode of processing in the RH has also been studied by cross-hemispheric presentation of word stimuli at a short SOA (e.g., Abernethy and Coney, 1996; Korsnes and Magnussen, 2007). In a cross-hemispheric study, primes and targets are presented to opposite hemispheres (RVF prime – LVF target, LVF prime – RVF target). Therefore, presentation of a prime to the LH at a short SOA facilitates processing of a target subsequently presented to the RH, whereas presentation of a prime to the RH does not facilitate processing of a target subsequently presented to the LH.

## Appendix: Stimulus Lists

**Table A1:** Stimulus pairs in the first and third lists including Negative–Negative pairs, Positive–Positive pairs, Negative–Positive pairs, and Positive–Negative pairs.

	Negative	Negative		Negative	Positive
(1)	HORROR	RIOT	(1)	PRISON	DOG
(2)	BURN	TRAITOR	(2)	ANGER	CAKE
(3)	FRAUD	BOMB	(3)	ULCER	PALACE
(4)	VICTIM	CRISIS	(4)	ACHE	DANCER
(5)	INSULT	JAIL	(5)	DECEIT	RESCUE
(6)	DEBT	LICE	(6)	TRAGEDY	FANTASY
(7)	SIN	HATRED	(7)	TORNADO	CASH
(8)	POISON	MENACE	(8)	HATE	VICTORY
(9)	ROBBER	SLAVE	(9)	COFFIN	HEAVEN
(10)	CANCER	MISERY	(10)	MALICE	PRIDE
(11)	QUARREL	DEVIL	(11)	FILTH	CIRCUS
(12)	INJURY	VENOM	(12)	HURT	WIN

	**Positive**	**Positive**		**Positive**	**Negative**

(1)	TREAT	SCHOLAR	(1)	GIFT	SLIME
(2)	LEADER	MIRACLE	(2)	GLORY	TORTURE
(3)	HOLIDAY	BABY	(3)	PASSION	ASSAULT
(4)	PROFIT	FUN	(4)	DINNER	THIEF
(5)	LIBERTY	TALENT	(5)	THRILL	AGONY
(6)	DESIRE	SONG	(6)	SNOW	HELL
(7)	KISS	TRAVEL	(7)	SUCCESS	CRIME
(8)	JOY	STAR	(8)	PUPPY	SCORN
(9)	COMEDY	DELIGHT	(9)	DIAMOND	MEASLES
(10)	DOLLAR	TRIUMPH	(10)	BLOSSOM	BURIAL
(11)	TOY	GOLD	(11)	LUST	DEMON
(12)	BOUQUET	JOKE	(12)	KING	LIE

*Within each list, half of the pairs were presented to the RVF and half to the LVF. The two lists were different in terms of the visual field of presentation.*

**Table A2:** Stimulus pairs in the second and fourth lists including Negative–Negative pairs, Positive–Positive pairs, Negative–Positive pairs, and Positive–Negative pairs.

	Negative	Negative		Negative	Positive
(1)	PRISON	SLIME	(1)	HORROR	SCHOLAR
(2)	ANGER	TORTURE	(2)	BURN	MIRACLE
(3)	ULCER	ASSAULT	(3)	FRAUD	BABY
(4)	ACHE	THIEF	(4)	VICTIM	FUN
(5)	DECEIT	AGONY	(5)	INSULT	TALENT
(6)	TRAGEDY	HELL	(6)	DEBT	SONG
(7)	TORNADO	CRIME	(7)	SIN	TRAVEL
(8)	HATE	SCORN	(8)	POISON	STAR
(9)	COFFIN	MEASLES	(9)	ROBBER	DELIGHT
(10)	MALICE	BURIAL	(10)	CANCER	TRIUMPH
(11)	FILTH	DEMON	(11)	QUARREL	GOLD
(12)	HURT	LIE	(12)	INJURY	JOKE

	**Positive**	**Positive**		**Positive**	**Negative**

(1)	GIFT	DOG	(1)	TREAT	RIOT
(2)	GLORY	CAKE	(2)	LEADER	TRAITOR
(3)	PASSION	DANCER	(3)	HOLIDAY	BOMB
(4)	DINNER	PALACE	(4)	PROFIT	CRISIS
(5)	THRILL	RESCUE	(5)	LIBERTY	JAIL
(6)	SNOW	FANTASY	(6)	DESIRE	LICE
(7)	SUCCESS	CASH	(7)	KISS	HATRED
(8)	PUPPY	VICTORY	(8)	JOY	MENACE
(9)	DIAMOND	HEAVEN	(9)	COMEDY	SLAVE
(10)	BLOSSOM	PRIDE	(10)	DOLLAR	MISERY
(11)	LUST	CIRCUS	(11)	TOY	DEVIL
(12)	KING	WIN	(12)	BOUQUET	VENOM

*Within each list, half of the pairs were presented to the RVF and half to the LVF. The two lists were different in terms of the visual field of presentation.*
